# Culinary Home Empowerment for Food Waste Prevention and Minimization: Feasibility and Efficacy Protocol

**DOI:** 10.3390/foods13162529

**Published:** 2024-08-14

**Authors:** Brandy-Joe Milliron, Roni Neff, Rachel Sherman, DeAndra Forde, Lauren Miller, Dahlia Stott, Alison Mountford, Jonathan M. Deutsch

**Affiliations:** 1 Department of Health Sciences, College of Nursing and Health Professions, Drexel University, Philadelphia, PA 19104, USAdps85@drexel.edu (D.S.); st96d633@drexel.edu (J.M.D.); 2 Department of Environmental Health & Engineering, Bloomberg School of Public Health, Johns Hopkins University, Baltimore, MD 21218, USA; 3 Drexel Food Lab, Department of Health Sciences, College of Nursing and Health Professions, Drexel University, Philadelphia, PA 19104, USA; rms548@drexel.edu (R.S.); lem324@drexel.edu (L.M.);

**Keywords:** food loss and waste, sustainable development, pedagogy, kitchen literacy, culinary education, public health, community based, consumer

## Abstract

The purpose of this research is to evaluate the feasibility, acceptability, and preliminary efficacy of a household food-waste prevention and minimization intervention, titled the Culinary Home Empowerment for Food Waste Prevention and Minimization (CHEF-WPM), which consists of a culinary education video series for home cooks. The specific aims are to (1) assess the effects of the intervention at a population level across process (feasibility, usage, acceptability, satisfaction) and preliminary efficacy (motivation, opportunity, ability) metrics and (2) assess the effects of the intervention at a community level across process (feasibility, usage, acceptability, satisfaction) and preliminary efficacy (motivation, opportunity, ability, household food waste, sustainable dietary practices) metrics. The intervention includes eight modules, each containing three to five brief videos, as well as downloadable recipes and worksheets. The evaluation will explore the effects of the program through two distinct investigations, namely (1) voluntary access to the intervention content in a population-based setting and (2) intensive delivery of the intervention content as part of a remote class in a community setting. Evaluation of the intervention in the population-based setting will use a single-arm, quasi-experimental post-test only study design. All home cooks who access the videos will be invited to answer a five-question post-video survey about acceptability, satisfaction, and potential implementation of the learning. A separate sample of individuals will be recruited to participate in a more in-depth evaluation (pre- and multiple post-test survey). Evaluation of the community-based intervention will use a mixed methods study design. Findings from the two distinct evaluation studies will be jointly discussed and triangulated to support larger conclusions about the intervention’s desirability, impact on motivation, opportunity, ability, and food waste, and the potential directions for further improvement.

## 1. Introduction

As the global population grows, the demand for food is projected to increase by over 50% in the next 50 years, assuming current eating patterns remain unchanged [1]. According to the United Nations Environment Programme (UNEP), one-third of all food produced worldwide is lost or wasted annually [2]. Food waste is the largest component of landfills and incineration streams [3], with the majority occurring at the consumer level [2]. In the U.S., over 60% of food waste occurs at the consumption stage, including households and restaurants [3]. This significant amount of wasted food presents opportunities to combat food insecurity, save energy, and address environmental damage and climate change [3].

Food waste in high-income countries is primarily attributed to consumer behavior and policy. The National Academy of Sciences (NAS) has identified 11 consumer-level drivers of food waste based on the Motivation–Opportunity–Ability (MOA) framework [4]. These drivers include consumer knowledge and skills, goals related to food and nutrition, food preferences and diet, psychological distance from food, food environments, and relevant policies and regulations [4].

Block et al.’s four stages to consumer decisions align closely with the NAS’s consumer-level drivers to food waste, as each stage is significantly influenced by consumer knowledge, skills, and food environments [5]. For example, in the *pre-acquisition stage*, consumer knowledge regarding the safe handling and culinary use of blemished produce can determine whether it will be purchased and used or discarded. Similarly, in the *acquisition stage*, consumers’ ability to accurately calculate purchase amounts helps prevent overbuying food products. During the *consumption stage*, household food environments influence opportunities to avoid food waste by practicing proper food and leftover storage. Finally, in the *disposition stage*, consumer knowledge of food conservation and sustainable practices can reduce a household’s food-waste impact [4,6].

### 1.1. The Consumer Food-Waste Paradox

Despite expressing a dislike for wasting food, consumers often engage in behaviors that result in food waste. To explore this paradox, Le Borgne et al. evaluated consumer concern for food waste and its impact on adopting “waste-prevention routines” [7]. They identified two dimensions of consumer concern: “individual/interpersonal concern”, which strongly influenced waste-prevention behaviors, and “global concern”, which did not have a significant effect. Their findings suggest that effective strategies to reduce household food waste should focus on enhancing individual/interpersonal concerns through economic concerns, food involvement, and food education. Similarly, Romani et al. emphasized the importance of “routine food management” behaviors in preventing food waste and highlighted the potential of targeted educational programs to promote sustainable food practices [8]. Their research indicates that daily food practices (shopping, cooking, and eating) and key behaviors (over-purchasing, poor meal-planning, and improper food storage) significantly contribute to household food waste and should be the focus of educational interventions.

### 1.2. Theoretical Underpinnings of Household Food-Waste Prevention

The Theory of Planned Behavior (TPB) [9] and Triandis’ Theory of Interpersonal Behavior (TIB) [10] have been used to study consumer and household food-waste behaviors. TPB posits that behavior is driven by *attitudes* (e.g., a belief that wasting food is bad for the environment), *subjective norms* (e.g., societal expectations about food waste), and *perceived behavioral control* (e.g., self-efficacy in being able to reduce food waste). Triandis’ TIB expands on TPB by integrating *habit* (e.g., habitual over-purchasing of food), *emotions* (e.g., guilt associated with wasting food), and *facilitating conditions* (e.g., availability of food storage facilities). By integrating these theories into a comprehensive framework, interventions can address both the rational and habitual aspects of food-waste behavior.

**Figure 1:**
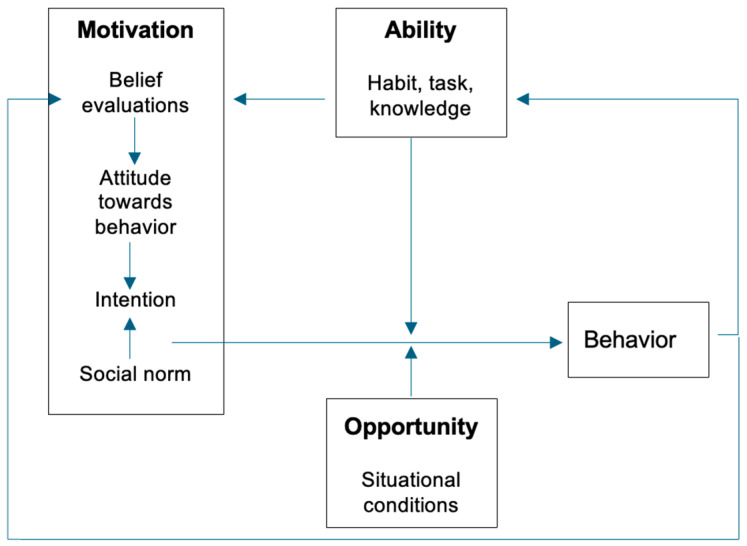
The Motivation–Opportunity–Ability Model.

The Motivation–Opportunity–Ability (MOA) Model has been viewed as an integration of the Theory of Planned Behavior with Triandis’ Theory of Interpersonal Behavior [11]. The MOA Model offers a targeted framework for understanding and influencing consumer behavior by considering *motivation*, an individual’s desire or willingness to engage in a behavior (e.g., household food-waste prevention); *opportunity*, external circumstances that enable or inhibit the behavior (e.g., access to tools and resources that make it easier to reduce food waste, such as meal planning tools, food storage containers, kitchen utensils); and *ability*, an individual’s capacity to perform the behavior (e.g., skills and knowledge to reduce food waste in the kitchen) (Figure 1) [11]. Adoption of the target behavior builds self-efficacy and reinforces ability and motivation, creating a positive feedback loop. A signature feature of the MOA Model is its integration of motivational, habitual, and contextual factors into a single model that can be used to explain pro-environmental behavior [11].

### 1.3. Cooking Education as a Tool for Reducing Household Food Waste

Cooking education is a crucial tool for reducing household food waste. It enhances individuals’ food agency—the ability to purchase and prepare food within their social, physical, and economic environments—empowering them to achieve their food-related goals [12]. Studies have shown that cooking interventions promote good nutrition and sustainable food practices [13,14,15,16]. Countries like Finland and Australia have incorporated home economics into educational curricula to mitigate food waste and enhance household sustainability [17]. Home economics educates consumers to be thrifty and mindful with food, make informed decisions about resources, and understand the environmental impact of their actions [18].

### 1.4. Specific Aims

While the NAS study provides a host of suggestions to reduce household food waste [4], translating the aforementioned findings into practice at the population and community levels is beyond the scope of most studies. Hence, the protocol described herein tests an approach to translating promising scientific findings into consumer-facing food-waste prevention and minimization lessons. The purpose of this protocol paper is to detail the design and methodology of a household food-waste prevention and minimization intervention titled Culinary Home Empowerment for Food Waste Prevention and Minimization (CHEF-WPM). Consistent with a protocol paper, this paper was submitted before data collection and, as such, does not present results or statistical analysis. Follow-on papers after data collection will do so [19]. The intervention consists of a culinary education video series for home cooks. The aims of the study are to:

Aim 1: assess the effects of CHEF-WPM at a population level across process (feasibility, usage, acceptability, satisfaction) and preliminary efficacy (motivation, opportunity, ability) metrics;

Aim 2: assess the effects of CHEF-WPM at a community level across process (feasibility, usage, acceptability, satisfaction), preliminary efficacy (motivation, opportunity, ability, household food waste), and exploratory (sustainable dietary behaviors) metrics.

It is valuable to study outcomes from both online exposures to the videos and outcomes from a more intensively implemented community-level delivery to gain insights into the effects of different types of deployment. Such insights could shape future motivation–opportunity–ability-oriented efforts using these or other videos.

## 2. Methods

The evaluation will explore the effects of the program through two distinct investigations, namely (1) when the intervention content is accessed voluntarily in a population-based setting (population CHEF-WPM) and (2) when the intervention content is accessed through intensive delivery as part of a remote class in a community setting (community CHEF-WPM). The population CHEF-WPM investigation will include two types of evaluations, and the community CHEF-WPM will include one type of evaluation. The subsequent sections in this report, as applicable, will be divided into the following three sections for clarity: *Population Intervention 1*, *Population Intervention 2*, and *Community Intervention*. This protocol is reported in accordance with SPIRIT (Standard Protocol Items: Recommendations for Interventional Trials) guidance. A completed SPIRIT checklist is available in Additional File 1. Additionally, Table 1 displays an overview of the objectives and methods for the three distinct evaluation components of the CHEF-WPM intervention.

### 2.1. Intervention

The overall goal of the CHEF-WPM intervention is to increase consumers’ motivation, perceived opportunity, and ability to reduce food waste using home-based culinary techniques. The intervention will include eight modules, each containing three to five brief (4–8 min) videos, as well as downloadable recipes and worksheets. CHEF-WPM is designed to be used *a la carte* or as a self-paced online course.

The eight module topics include:

1. My whole plate: Meal Planning & Kitchen Management with a ‘No Food Waste’ Lens;
2. Veggies: Roots to Stem;
3. Fruit: Fresh or Bruised;
4. Meat- & Plant-Based Proteins;
5. Fish & Seafood: Sear It, Don’t Fear It;
6. Bread, Grains, Snacks, Sweets;
7. Cooking Liquids & Dairy;
8. Leftovers Are My Jam.

**Figure 2:**
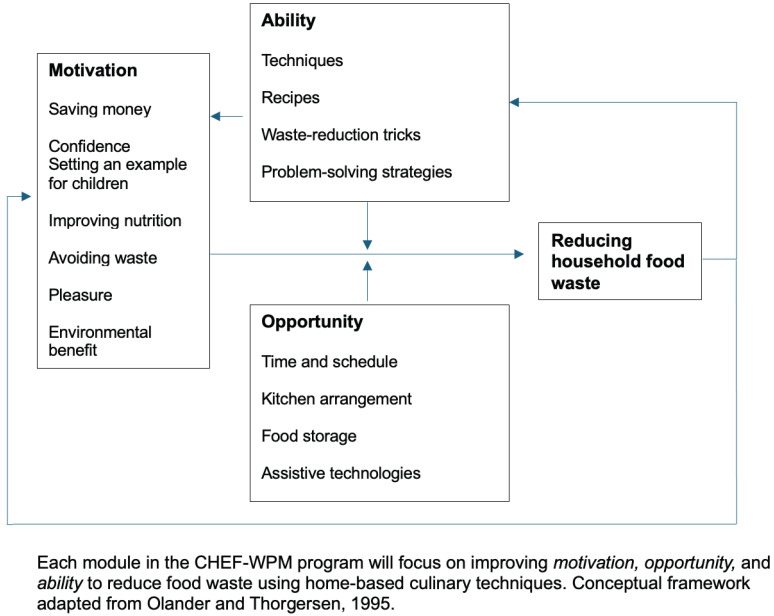
Conceptual framework of CHEF-WPM program [11].

Each module focuses on improving the *motivation*, *opportunity*, and *ability* to reduce food waste using home-based culinary techniques. The modules will highlight *motivations*, including saving money, avoiding waste, confidence, setting an example for children, and improving nutrition, pleasure, and environmental benefits. The modules will also focus on perceived *opportunity* and illustrate ways to shift contextual factors, such as time and schedule, kitchen arrangement and food storage, and technologies, among other aspects. *Ability* will be addressed via sharing techniques, recipes, and tricks to prevent and minimize food waste at home and problem-solving strategies for challenges to food waste prevention. Adapted from the work of ölander and ThØgersen [11], Figure 2 displays the conceptual framework guiding the CHEF-WPM program.

The content will be developed and filmed at Drexel’s College of Nursing and Health Professions’ Food and Hospitality Management Space, and in the kitchen studio of the food-waste educator. The developed content will be hosted on a freely accessible website, Kajabi (http://kajabi.com; accessed on 1 July 2024). The CHEF-WPM program will be built and delivered to key audiences as an online course on Kajabi. Kajabi is an “all-in-one” platform that we will use to build our program, engage with learners (participants), and collect feasibility and preliminary efficacy metrics. The videos will also be disseminated via social media (i.e., X [FKA Twitter], Instagram, TikTok) and through partnerships with food-security organizations, retailers, nutrition education programs, and others.

### 2.2. Population Intervention 1

#### 2.2.1. Overview

The evaluation of Population Intervention 1 will use a single-arm post-test only study design. All home cooks who access the videos will be invited to answer a brief post-video survey about acceptability and satisfaction (*n* = 1068).

#### 2.2.2. Study Population and Recruitment

Potential participants will be 18 years or older, responsible for at least half of their household food preparation, prepare meals at home at least twice each week, have access to the internet, and understand English. The following groups will be excluded: adults unable to consent, individuals who are not yet adults (infants, children, and teenagers), and prisoners. Participants will be recruited through Drexel Food Lab networks and in partnership with community and national organizations. Recruitment methods will include digital and paper advertisements, email advertisements, QR codes, and other methods, such as shelf-tags at local libraries. QR codes and email links will direct individuals to the CHEF-WPM videos. At the end of each video, the viewers will have the opportunity to “opt-in” to complete a short survey to assess satisfaction, engagement, and comprehension. Our goal is to recruit a minimum of 1068 individuals to participate in that brief survey, providing 95% power to detect an effect size of 0.3. However, we will collect and analyze data from all of the participants who complete the 5-question survey. In other words, *we will not cease recruitment* once we obtain a sample size of 1068 quality answers.

#### 2.2.3. Data Collection and Outcomes

As described, all home cooks who access the videos will be invited to answer a brief post-video survey about acceptability and satisfaction. At the end of each video, viewers will have the opportunity to “opt-in” to complete a short survey to assess satisfaction, engagement, and comprehension. If they agree, they will be redirected to a study information sheet and screening questionnaire. If they fit the study’s inclusion criteria, they will proceed to a brief survey via Qualtrics that will assess satisfaction, engagement, and comprehension of the video content. The brief survey will take less than 2 min to complete.

### 2.3. Population Intervention 2

#### 2.3.1. Overview

The second type of evaluation in the population-based setting will use a single arm, quasi-experimental pre- and multiple post-test study design. A sample (*n* = 500) of participants will be recruited to participate in a ~10 min survey to evaluate three specific videos across process (feasibility, satisfaction, engagement, comprehension) and preliminary outcome (motivation, opportunity, ability) metrics. The participants will be recontacted again at 3 and 6 months to complete follow-up surveys about longer-term outcomes.

#### 2.3.2. Study Population and Recruitment

The inclusion and exclusion criteria are the same for both population-level interventions. Potential participants should be 18 years or older, responsible for at least half of their household food preparation, prepare meals at home at least twice each week, have access to the internet, and understand English. The following groups will be excluded: adults unable to consent, individuals who are not yet adults (infants, children, and teenagers), and prisoners.

We will recruit participants for this evaluation by utilizing ResearchMatch and Build Clinical. ResearchMatch is a website that connects potential participants with researchers by emailing individuals with a recruitment message that explains the basic details of the research study. If individuals are interested in participating in the study, they will be directed to a screening questionnaire. Build Clinical will run an online advertisement campaign on online and social media websites, such as Facebook, Instagram, and X (formerly Twitter). When individuals press on these digital advertisements, they will be redirected to a landing page where they can learn more about the study and fill out a screening questionnaire.

If the interested individual from either platform is eligible for the study, a research assistant will call the individual and further explain the goals and procedures of the intervention. After this phone call, the research assistant will send the individual an email with a link to the information sheet for exempt research. Proceeding to the survey will serve as a proxy for written consent. Participants will be offered a USD 10 incentive for completion of the pre- and post-video surveys, and an additional USD 5 incentive for completion of each follow-up assessment (3 and 6 months; USD 10 for completion of both; USD 20 for completion of the entire study).

A sample size of 500 is sufficient to provide 95% power to detect an effect size of 0.43. Participants will be purposively sampled by age, race/ethnicity, gender, and income to optimize the diversity of our sample. Our goal is for our sample to mirror the US census demographic composition of the most recent census (Gender—Male: 49.6%, Female: 50.4%, Non-binary: natural fallout; Race—White: 76%, Black/AA: 14%, Asian or Pacific Islander: 6%, American Indian/Alaskan Native/Other: 2%; Hispanic Ethnicity—Hispanic: 19%, Non-Hispanic: 59%).

#### 2.3.3. Data Collection and Outcomes

A structured survey (~10-min pre-post survey) will evaluate specific educational videos across process (feasibility, usage, acceptability, and satisfaction) and preliminary outcome (motivation, opportunity, and ability) metrics. The enrolled participants will also complete 3- and 6-month follow-up surveys. The structured survey will ask about demographic characteristics and will obtain baseline information about motivation, opportunity, and ability as they relate to food-waste prevention. The survey will also contain three embedded food-waste prevention videos for the participants to view. After viewing each video, the participants will be asked to answer questions about the content to assess video satisfaction, engagement, recall, and comprehension of information, in addition to questions about motivation, opportunity, and ability. The 3- and 6-month follow-up surveys will primarily focus on motivation, opportunity, and ability to reduce household food waste.

#### 2.3.4. Data Analyses for Population Interventions 1 and 2

Descriptive characteristics of our study sample will be calculated as means ± standard deviations, frequencies, chi-square contingency analyses, and standardized residuals, unless otherwise noted. Changes in motivation, opportunity, and ability will be analyzed as continuous or categorical variables. Mixed factorial analyses of covariance (ANCOVA) with repeated measures will be used to determine whether mean differences exist in participant satisfaction, engagement, and comprehension after controlling for the influence of socio-demographic- (age, race and ethnicity, marital status, education, and income) and household-related characteristics measured at baseline. Differences will be estimated for all categorical variables using McNemar’s exact significance probability testing for paired nominal data. All quantitative statistical analyses will be accomplished using IBM SPSS Statistics.

### 2.4. Community Intervention

#### 2.4.1. Overview

All eight modules of the CHEF-WPM intervention will be presented in sequence over an eight-week period, facilitated by a chef–instructor with assistance from culinary students. The course will include synchronous discussions and group viewing of the CHEF-WPM video series hosted on Kajabi. Evaluation of the remote community-based intervention will use a mixed methods evaluation study design. The objectives of this mixed methods evaluation are to assess (1) intervention implementation (fidelity), satisfaction, engagement, and comprehension; (2) intervention outcomes (motivation, opportunity, ability, and household food waste); and (3) exploratory outcome (sustainable dietary behaviors). A maximum of 60 participants will be recruited in partnership with local community organizations for quantitative data-collection activities. Quantitative data collection will occur pre- and post-8-week intervention and at 6 months follow-up. A purposive sample of 20 participants will be recruited to participate in in-depth interviews at 8 weeks post-intervention. Food waste will be measured via self-report methods among the participants (*n* = 60) at pre- and post-intervention and 6-month follow-up.

#### 2.4.2. Study Population and Recruitment

Potential participants will be 18 years or older, responsible for at least half of their household food preparation, prepare meals at home at least twice each week, have access to the internet, and understand English. The following groups will be excluded: adults unable to consent, individuals who are not yet adults (infants, children, and teenagers), and prisoners. Individuals who express interest in participating in the 8-week CHEF-WMP program will be screened for eligibility via telephone or a brief Qualtrics survey.

A convenience sample of 60 participants will be recruited for the community-level intervention, drawn primarily from a lower-income population in Philadelphia (3 cohorts of 20 individuals per cohort). This population includes a higher percentage of Black, Indigenous, and people of color than the US population. For the qualitative evaluation (in-depth interviews), a purposive sample (*n* = 20) of intervention participants will be recruited. Purposive sampling helps ensure sample diversity across age, income, educational attainment, household size, and baseline pre-disposition for food waste prevention. Participants will be recruited through university networks and in partnership with community organizations. Participants will be recruited via phone calls and/or email, digital and paper advertisements, and QR codes. These organizations together generally attract participants reflecting the city’s diverse urban demographics, namely 44% Black, 13% Latino, 7% Asian; 46,000 median household income; and 20% poverty rate.

QR codes and email links will direct individuals to a Qualtrics screening questionnaire. If they qualify, they will proceed to a study information sheet that describes participation in detail. If they are still interested after learning more about participation, they will be asked to submit their contact information and a research assistant will contact them with further details about participation. Participants will be compensated a total of USD 150 for completing the evaluation activities (quantitative measures) and an additional USD 40 for completing an interview.

#### 2.4.3. Data Collection and Outcomes

The participants will complete a pre-intervention survey and measure their household food waste before beginning the 8-week intervention. At the completion of the intervention, participants will complete a post-intervention survey and again measure their household food waste. A purposive sample of participants, six from each cohort, will be recruited to complete in-depth interviews at the end of the intervention. We will follow up with all the participants 6-months after the intervention, where they will complete a 6-month post-intervention survey and measure their household food waste. These measures are described below.

For the *pre- and post-intervention surveys*, after viewing the study information page for exempt research, the participants will proceed to a structured survey that will ask about demographic characteristics and will obtain baseline information about motivation, opportunity, and ability as they relate to food-waste prevention. To the extent possible, the evaluation questions were replicated from existing surveys and tailored to this intervention, including the specific content of each media asset. After completing the pre-intervention survey, the participants will be provided a scale and food-waste diary and given a brief training by a study researcher on measuring their home food waste. They will keep a one-week food-waste diary, as described below. The participants will be asked to complete a brief satisfaction survey after each program session.

The post-intervention assessment will occur at the end of the 8-week program. A survey that includes the baseline *survey* questions and additional post-intervention questions will be sent to participants during the one-week period after their last intervention session. In this post-intervention survey, the questions will assess program satisfaction, engagement, recall, and comprehension of information, participants’ level of implementation of steps recommended in the videos, further engagement with and shares of videos, reactions to the intervention, and intention to continue engaging in food-waste prevention activities. Participants will again receive instructions on how to measure their food waste for one week. The 6-month follow-up assessment will include a similar survey and food-waste measurement.

For *household food waste*, the participants will measure their household food waste for one week via self-report before and after participating in the remote community-based class. The participants will be given a Taylor compact digital scale and trained on how to measure their food waste. Our team will create a training video and instructions for how to measure food waste at home and will provide visual reminders for key aspects of the measurement, such as what to classify as “avoidable” vs. “unavoidable”. The participants will be given a diary form and instructed that, before they or a household member discards any household food waste to any destination including the sink and whether it is trim from meat or produce, plate waste, prepared leftovers, spoiled food, etc., they should weigh the food, describe what it is (fruit, vegetable, cooked grain, etc.), and indicate its type (trim from cooking, spoiled food, leftovers, plate waste, or other), as well as their impression of whether the food waste was unavoidable (example, fish innards) or avoidable (example, spoiled milk from overbuying) through a simple coding system, taking a minute or less. This requires discussion and buy-in from household members. The participants will again be asked to participate in the food-waste assessment at the 6-month follow-up.

For *sustainable dietary practices*, at pre- and post-intervention, and the 6-month follow-up, the participants will be asked to complete a brief dietary assessment to measure the overall sustainability of their dietary practices. This assessment will be conducted using Diet ID via their metric called Dietary Impacts on Environmental Measures (DIEM). The DIEM metric helps us understand the environmental footprint of participants’ diets. Diet ID is a novel pattern-recognition-based method that facilitates fast, user-friendly, and scalable dietary assessment [20]. Participants will be prompted with two photos of diets and asked which of the two is more like what they usually eat. Once they have chosen, the participants will be prompted with another two photos and asked the same questions. Participants will answer questions about their gender, height, weight, age, dietary restrictions/allergies, and general physical activity level (low, moderate, high) before completing the dietary assessment. No identifiable information will be collected by Diet ID.

For *qualitative data collection*, a purposive sample (*n* = 20) of intervention participants will be recruited to maximize diversity across age, income, educational attainment, household size, and baseline pre-disposition for food-waste prevention. They will participate in in-depth interviews after intervention completion to evoke priorities, values, and wisdom related to food-waste prevention; reactions to the intervention; and effects on motivations, opportunities, and abilities, as well as barriers and facilitators for continued engagement in food-waste prevention behaviors, and potential co-benefits for other outcomes as well as undesired tradeoffs and spillovers. Interviews will be led by an experienced qualitative researcher, will be conducted on HIPPA-compliant Zoom, will last approximately 30–45 min, and will be audio-recorded and transcribed verbatim.

#### 2.4.4. Data Analyses

Descriptive characteristics of our study sample will be calculated as means ± standard deviations, frequencies, chi-square contingency analyses, and standardized residuals, unless otherwise noted. Changes in food-waste prevention behaviors, discards, motivation, opportunity ability, potential economic and nutrition-related savings, and DIEM score will be analyzed as continuous or categorical variables. Mixed factorial analyses of covariance (ANCOVA) with repeated measures will be used to determine whether mean differences exist in participant satisfaction, engagement, comprehension, changes in knowledge and planned behaviors, and food-waste variables based on socio-demographic (age, race and ethnicity, marital status, education, and income) and household-related characteristics measured at baseline. Differences will be estimated for all categorical variables using McNemar’s exact significance probability testing for paired nominal data. All quantitative statistical analyses will be accomplished using IBM SPSS Statistics. As the purpose of proof-of-concept studies is to determine whether the intervention merits more costly testing using a randomized controlled trial design, small sample sizes are acceptable because clinical, not statistical, benefit is sought.

For qualitative data, the interviews will be analyzed using content analysis based on a deductive–inductive approach. First, interview segments related to the drivers of food-waste prevention (motivation, opportunity, and ability) will be identified. For each driver, themes will be identified inductively. 

In this study, once the quantitative and qualitative data are analyzed separately, the data will then be merged together to have a greater understanding of the effects of the intervention. This will allow discongruence and congruence between both sets of data to emerge. We will report the mixed methods results with joint displays.

#### 2.4.5. Community Advisory Board

A community advisory board has been recruited from our community partners to provide input into the design, content, guest-chef representation, and recipes of the videos. The advisory board will meet quarterly via Zoom and in-person (hybrid model), and each member will be paid an incentive for their participation.

### 2.5. Withdrawal

The study team does not anticipate circumstances under which participants would need to be withdrawn from the research without their consent. Participants may elect to leave the study at any time with no penalty. If a withdrawn participant completed the study survey, their data may still be included in our analysis. Additionally, if participants withdraw from the study before the end of data collection, they will not be compensated.

## 3. Potential Benefits and Risks of Study Participation

As with any study involving the collection of data, there is a risk of harm resulting from a breach of privacy and/or confidentiality. If information linking participants to their responses is inadvertently disclosed, we do not anticipate any significant harm to result to participants. However, we will still secure the information we collect (as outlined in the data-protection section) to minimize the probability of this risk. We will also maintain feedback and discussion data in separate folders without individual identifiers. Once the research study is complete and individually identifiable information is no longer necessary, the key linking individuals to their responses will be destroyed, further reducing the magnitude of this risk.

During the interviews, it is possible that some activities or questions will lead to momentary embarrassment, discomfort, or unease. The probability and magnitude of discomfort are not expected to be greater than participants would ordinarily encounter in their everyday lives and will be disclosed during the informed consent process. We do not anticipate any costs to participants for taking part in this study. We do not expect any of our procedures will carry unforeseen risks to participants or individuals not participating in the research.

There may be no direct benefit to participants of this research study, though they may value the opportunity to participate in the class. The information collected may benefit society by developing a novel intervention to enhance participants’ motivation, opportunity, and ability to reduce consumer food waste. There are minimal risks associated with this study. The probability and magnitude of discomfort are not expected to be greater than participants would ordinarily encounter in their everyday lives and will be disclosed during the informed consent process. The study procedures, particularly the food-waste tracking, require an investment of time and effort.

## 4. Data Monitoring

The principal investigators will monitor and review the study progress, participant safety, and the accuracy and security of the emerging data. All information collected in this study will be kept confidential as required by law. Names will not be attached to the study participants and a master list, and the data elements and survey responses will be kept in a secure and encrypted database, which can only be accessed by research team members. Due to the low-risk nature of the intervention, a Data and Safety Monitoring Board has not been formally established. All adverse events will be reported to the principal investigators and reviewed by the study team to determine whether the study should be discontinued due to participant safety. Quality assurance procedures will be implemented, including rigorous attention to manual data cleaning, generation of monthly query reports, and ranges and data validation checks.

## 5. Ethics and Dissemination

### 5.1. Consent

The consent process for each of the three types of evaluations will be the same. The participants will complete a screening questionnaire to determine study eligibility. If eligible, they will be directed to a study information sheet for exempt research. After reading the information sheet for exempt research, proceeding to the survey will serve as a proxy for informed consent. In the information sheet, the research team’s contact information will be provided to the participant. Participants are encouraged to contact the research team if there are any questions and will be given the opportunity to discuss the study with their surrogates or think about it prior to agreeing to participate. They may withdraw consent at any time throughout the study. A copy of the information sheet will be emailed to participants for their records.

### 5.2. Confidentiality

Participant confidentiality is strictly held in trust by the investigators, study staff, and the sponsor and their agents. The study protocol, documentation, data, and all other information generated will be held in strict confidence. No information concerning the study or the data will be released to any unauthorized third party without prior written approval of the sponsor. The study monitor or other authorized representatives of the sponsor may inspect all study documents and records required to be maintained by the investigator. 

Confidentiality will be protected by including only information needed to assess study outcomes, minimizing to the fullest extent possible the collection of any information that could directly identify participants, and maintaining all study information in a secure manner. To ensure participant privacy and confidentiality, a unique study identifier will be assigned to each participant. The data and records will be kept locked and secured, with any computer data password protected. Protected health information (PHI) will not be collected in this study. Therefore, we are requesting a waiver of HIPAA authorization. To ensure privacy, all interviews will be conducted via HIPAA-compliant Zoom technologies. Links to Qualtrics surveys will be encrypted. To protect the confidentiality of participant responses, the data will be de-identified and unique identification numbers will be assigned to participants.

### 5.3. Dissemination

We will disseminate the study findings through conference presentations and publications in peer-reviewed international journals. Only aggregate data without individually identifiable information will be published. As this study is a feasibility study, the results will be used to inform and develop a larger, adequately powered, randomized controlled trial, if appropriate.

## 6. Results

This research investigates a highly scalable solution to strengthen consumers’ motivation, opportunity, and ability to reduce food waste at the locus where most wasted food occurs by adapting professional culinary education interventions to the home kitchen. This interdisciplinary project combines knowledge from the fields of culinary arts and science, education, consumer behavior, nutrition, environmental science, and public health to provide low-friction interventions and flexible access to content that will resonate with consumers. Rather than teaching home cooks through an overbearing and expensive didactic approach, this intervention empowers home cooks to prevent and minimize food waste in a way that is convenient, accessible, culturally appropriate, and scalable. This intervention offers home cooks the opportunity for professional-level culinary education in a way that saves money, utilizes cultural flavors, provides nutrition background information, and builds confidence, all while reducing food waste.

This research also includes multiple innovative features, addressing critical research gaps and pioneering new approaches:

1. *Cooks as consumer educators*, reflecting the NAS report highlighting the need to use such influencers [4];
2. *Bite-size videos*, adapting food waste education to a social media-friendly modality that many people use to learn about cooking today. This allows us to create or support new or existing influencers, whose reach and appeal are hard to match through more traditional research interventions;
3. *Innovative application and assessment* of motivation, opportunity, and ability (MOA) metrics [11];
4. *Innovation in a longer timeframe of follow-up* and in comparing and contrasting the same intervention in both community and population settings;
5. *Direct measurement of waste*.

## 7. Discussion

Wasted food is the largest component of the landfill and incineration stream [3], and most of the waste generated remains at the consumer level [2]. Hence, the purpose of this research protocol is to evaluate the feasibility, acceptability, and preliminary efficacy of a household food-waste prevention and minimization intervention, which consists of a culinary education video series for home cooks. The specific aims of this protocol are to (1) assess the effects of the intervention at a population level across process (feasibility, usage, acceptability, and satisfaction) and preliminary efficacy (motivation, opportunity, and ability) metrics and (2) assess the effects of the intervention at a community level across process (feasibility, usage, acceptability, and satisfaction) and preliminary efficacy and exploratory (motivation, opportunity, ability, household food waste, and sustainable dietary practices) metrics.

The evaluation seeks to understand intervention effects both when accessed voluntarily in a population-based setting, and via a more intensive delivery as part of a program in a community setting. Findings from the two distinct evaluation studies will be jointly discussed and triangulated to support larger conclusions about the intervention’s desirability, impact on motivation, opportunity, ability, and food waste, and the potential directions for further improvement. The intervention’s benefits may be both short and long term because the skills and attitudes presented can shape ongoing kitchen practices and norms.

### Final Considerations

Consistent with the protocol paper format, this paper does not present data but rather the methodology (protocol) that will be used to investigate the stated aims. We anticipate that the data analysis will help identify aspects of the intervention that are promising to prevent and minimize wasted food on a larger scale and will inform policy and funding for program interventions. Future publications will present the data and analysis from this protocol, and future protocols will test additional promising interventions, as suggested by our analysis in this study. If successful, the intervention will provide an affordable, scalable, and highly adaptable component that could be integrated into a national food-waste prevention campaign and other communication activities.

## Figures and Tables

**Table 1:** Overview of CHEF-WPM methodology.

Specific Aim	Setting	Study Design	Sample Size	Exposure	Outcomes
Aim 1: Assess the effects of CHEF-WPM at a population level across process and preliminary efficacy metrics.	Population intervention 1	Single-arm post-test only study design.	*N* ≥ 1068	All home cooks who access the videos will be invited to answer a brief post-video survey.	Satisfaction, engagement, and comprehension.
	Population intervention 2	Single arm, quasi-experimental pre- and multiple post-test study design (3- and 6-month follow-up).	*N* = 500	Three specific food waste videos that are embedded in surveys.	Feasibility, usage, acceptability, satisfaction, motivation, opportunity, and ability.
Aim 2: Assess the effects of CHEF-WPM at a community level across process, preliminary efficacy, and exploratory metrics	Community intervention	Mixed method evaluation with baseline, post-8-week intervention, and 6-month follow-up assessments. Qualitative data will be collected after 8-week intervention	*N* = 60	All eight modules of the CHEF-WPM intervention will be presented in sequence, over an eight-week period, facilitated by a chef-instructor.	Feasibility, usage, acceptability, satisfaction, motivation, opportunity, ability, household food waste, and sustainable dietary behaviors.

## Data Availability

The original contributions presented in the study are included in the article, further inquiries can be directed to the corresponding author.
